# Human trafficking and severe mental illness: an economic analysis of survivors’ use of psychiatric services

**DOI:** 10.1186/s12913-016-1541-0

**Published:** 2016-07-19

**Authors:** Maria Cary, Siân Oram, Louise M. Howard, Kylee Trevillion, Sarah Byford

**Affiliations:** King’s College London, David Goldberg Centre, De Crespigny Park, London, SE5 8AF UK

**Keywords:** Human trafficking, Electronic health records, Psychiatric case register, Secondary mental health services, Cost-analysis

## Abstract

**Background:**

Previous studies have found a high prevalence of depression and post-traumatic stress disorder (PTSD) among survivors of human trafficking. European countries are required to assist trafficked people in their psychological recovery, but there are no rigorous data on the costs of doing so. The objectives of this study were to quantify the use of secondary mental health services by survivors of human trafficking; to estimate the cost of survivors’ use of secondary mental health services provided by the UK National Health Service (NHS); and to identify factors that predict higher costs of mental health service provision.

**Methods:**

Historical cohort study of psychiatric patients who had experienced human trafficking. The South London and Maudsley NHS Trust (SLaM) Biomedical Research Centre Case Register Interactive Search (CRIS) database was used to identify anonymised full patient records of patients who had experienced human trafficking and who had accessed SLaM mental health services between 2007 and 2012. Data were extracted on socio-demographic and trafficking characteristics and contacts with mental health services. Total costs were calculated by multiplying each resource use item by an appropriate unit cost. Factors that predicted high mental health service costs were analysed using regression models.

**Results:**

One hundred nineteen patients were included in the analysis. Mean total mental health service costs per patient were £27,293 (sd 80,985) and mean duration of contact with services was 1490 (sd 757) days (approximately 4 years). Regression analysis showed that higher costs were associated with diagnosis of psychotic disorder (*p* < 0.001) and experiences of pre-trafficking violence (*p* = 0.06). Patients diagnosed with psychotic disorders cost approximately £32,635 more than patients with non-psychotic disorders/psychological distress but no formal diagnosis and patients whose clinical notes documented pre-trafficking violence cost £88,633 more than patients for whom pre-trafficking violence was not documented.

**Conclusions:**

Trafficked patients’ use of mental health services – and the cost of providing care – is highly variable, but patients with psychotic disorders and with experiences of pre-trafficking violence are likely to require more intensive support. Evidence is needed on the effectiveness of interventions to promote the recovery of survivors of human trafficking.

## Background

Human trafficking is the recruitment and movement of people – most often by force, coercion or deception – for the purposes of exploitation [1]. Exploitation may include forced sex work and labour in settings such as domestic work, agriculture, and construction. Research has shown a high prevalence of mental health problems among victims of human trafficking in contact with support services, including depression, anxiety and post-traumatic stress disorder [2–5], and has demonstrated that secondary mental health services in the UK are providing care for survivors of human trafficking with a range of diagnoses, including schizophrenia and related disorders [6, 7]. European law requires that governments assist victims of trafficking in their psychological recovery [8, 9], but to date there are no rigorous data on the likely costs of doing so.

This study addresses this evidence gap by providing robust estimates of trafficked people’s use of secondary mental health services and the associated cost to the UK National Health Service (NHS), and identifying factors that predict higher mental health service costs. The study uses data from a larger cohort study describing the socio-demographic, clinical, and service use characteristics of trafficked people in contact with secondary mental health services in South-East London, UK [6]. We hypothesised that the costs of mental health service use would be significantly higher among:

1. Trafficking survivors with a diagnosis of psychotic disorder, versus other diagnoses;
2. Trafficking survivors who experienced sexual exploitation, versus those who had experienced other forms of exploitation (e.g. domestic servitude, labour exploitation);
3. Trafficking survivors who experienced pre-trafficking violence, versus those who had not.

## Methods

### Study design

Historical cohort study of trafficked patients in contact with secondary mental health services.

### Setting

The study used data from the South London and Maudsley NHS Foundation Trust (SLaM) Biomedical Research Centre Case Register Interactive Search (CRIS) database [10]. SLaM provides secondary mental health services to the London boroughs of Croydon, Lambeth, Lewisham and Southwark (a catchment area of approximately 1.2 million people), and has a near 100 % monopoly on provision. The CRIS database allows the searching and retrieval of anonymised patient records for over 200,000 patients in contact with SLaM services.

### Participants

The study included SLaM service users whose clinical records indicated that they may have been trafficked for exploitation and who had one or more contact with SLaM services between 2007 and 2012. Trafficking was defined in accordance with the United Nations (UN) Optional Protocol to Prevent, Suppress and Punish Trafficking in Persons, Especially Women and Children (i.e. the recruitment or movement of people, by means such as force, fraud, coercion, deception, and abuse of vulnerability, for the purposes of exploitation), and included international and internal trafficking [1]. Trafficking search terms (see Supplementary Information) were used to search the free-text clinical notes and correspondence of all patients in contact with SLaM services during the study period and to retrieve the records of patients whose records included one or more of the search terms. One researcher assessed the returned records for eligibility in the study (records that documented concerns that the patient may have been trafficked as per the UN definition of human trafficking); a second researcher (SO) independently assessed the eligibility of 10 % of the records. There were three scenarios by which healthcare professionals became aware that their patient had been trafficked: (1) the patient disclosed their experiences of exploitation; (2) the patient presented with signs of abuse or exploitation that led the professional to suspect trafficking, or (3) the healthcare worker was informed by another professional (e.g. law enforcement, immigration, social services, voluntary sector, other health professionals) that their patient had been trafficked. Less detail regarding the type of exploitation was typically recorded in the third situation, but correspondence between professionals included other relevant information that indicated that the patient met the study criteria e.g. that the patient was involved in criminal proceedings against their trafficker, was claiming asylum in relation to their experiences while trafficked, or was receiving social services or voluntary sector support as a victim of trafficking.

### Data extraction and costing

Data were extracted on routinely recorded socio-demographic characteristics (e.g. gender, age, country of origin), clinical characteristics (e.g. International Classification of Disease-10 (ICD-10) diagnosis), mental health service characteristics (see Oram et al for full details [6]), and mental health service use. Mental health service use data included information on the date and duration of each contact, the type of professional contacted, the type of contact (inpatient, outpatient, accident and emergency or indirect contacts) and whether or not the patient attended. Data were also extracted from free-text clinical notes on patients’ experiences of physical and sexual violence prior to and during trafficking, and type of exploitation. Patients whose notes did not refer to violence prior to or during exploitation were categorised as not having experienced these types of abuse. Type of exploitation was categorised as sexual exploitation, domestic servitude, labour exploitation, financial exploitation (trafficking for benefit fraud, for example), or unknown.

Total costs were calculated by multiplying each resource use item by an appropriate unit cost. All unit costs, in United Kingdom (UK) pound sterling, were for the financial year 2012–2013 and included national NHS reference costs for hospital contacts [11] and national average unit costs for community health services [12]. No adjustments were made for inflation but costs were discounted to reflect time preferences. Costs were assumed to occur at the beginning of each year [13], and the discount rate used was 3.5 %, based on the recommendations of the UK Treasury for the discounting of costs [14] .

Indirect contacts (phone calls, letters, faxes and emails) were not costed as the cost of these contacts are included in the published unit costs through the use of appropriate direct to indirect contact ratios. The cost of appointments not attended were assumed to be equal to the full cost of the appointment, which assumes the professional involved failed to make productive use of the time. This assumption was reduced in sensitivity analysis to zero but this had little impact on the results presented, so only the main analyses are presented.

### Data analysis

Total costs over the period that each patient was in contact with SLaM services are presented as mean, standard deviation, median and range. Factors associated with total costs were explored using regression analysis. A list of possible cost predictors was created based on previous research investigating risk of mental health problems among trafficked people and in collaboration with clinical members of the research team [5, 15, 16]. This included: gender, age at first contact, diagnosis (psychotic disorder versus other), type of exploitation (sexual versus other [domestic servitude, financial exploitation, labour exploitation or unknown]), and violence pre- and during trafficking (sexual versus other). First, univariate associations between each of the specified predictors and total costs were explored in a linear regression. All variables are categorical except age at first contact, which is presented in two groups split at the median. In addition, age is presented split at the legal age for adulthood (<18 versus 18 and older) to assess any differences in the two populations. Secondly, multiple regression was used to reduce the variable set to those factors independently associated with mental health service costs. The model initially included all variables that had univariate associations with total costs at a significance level of 10 %, discarding from the model all variables that were no longer found to be important. Variables that did not have a univariate association were then added, one at the time, and retained if they added significantly to the model, otherwise discarded. The model derived was checked to ensure that no variables excluded would make a significant additional contribution [17]. To confirm the validity of this approach, multiple regression was used with all independent variables included.

Cost data are commonly skewed and as a result the choice of regression method is not straightforward. Although the ordinary least squares assumptions may be violated, namely linearity and homoscedasticity, it is not appropriate to transform costs as analysis is then not concerned with the arithmetic mean but with the geometric mean, which is of less value to decision makers [18]. For this reason, the results of the model were checked against the results obtained from a generalised linear model using an identity link function to describe the scale on which covariates in the model are related to costs and assuming a gamma distribution function for the costs [18]. Results were compared with the results from a non-parametric bootstrap regression in order to assess the robustness of the confidence intervals and *p*-values to non-normality of the cost distribution.

### Ethics and consent

Ethical approval for the research use of CRIS-derived anonymised databases without the written informed consent of SLaM service users was granted by an independent Research Ethics Committee (Oxfordshire C, reference 08/H0606/71). An Oversight Committee reviews all applications to use CRIS, and gave approval for this study (11/025).

## Results

A total of 119 patients were included in this analysis. The socio-demographic and clinical characteristics are summarised in Table 1 and are described in full elsewhere [6]. The majority of the sample were female (76 %, *n* = 91), and amongst them, two-thirds were trafficked for sexual exploitation (63 % of women). The median age at first contact with SLaM services was 22 years old (range from 8 to 49). The majority of the sample were diagnosed with non-psychotic disorders (72 %, *n* = 86). Psychotic disorders were present in just under a fifth of the sample (17 %, *n* = 20) and were more prevalent in men (39 %, *n* = 11) than women (10 %, *n* = 9). The remainder (11 %, *n* = 13) had psychological distress but no formal diagnosis (”psychological distress”). Violence prior to trafficking was documented in the records for almost half of the sample (48 %, *n* = 57), and violence during trafficking for 58 % (*n* = 69). Pre-trafficking violence was perpetrated by a variety of people, most commonly parents (14 %, *n* = 17), other family members (20 %, *n* = 24), and soldiers (10 %, *n* = 12), but also by acquaintances, strangers, teachers and pimps.

**Table 1:** Baseline characteristics by gender

Variable	Total (*n* = 119)	Male (*n* = 28)	Female (*n* = 91)
Type of exploitation			
Domestic servitude	17 (14 %)	1 (4 %)	16 (18 %)
Labour exploitation	8 (7 %)	8 (28 %)	0 (0 %)
Financial exploitation	4 (3 %)	0	4 (4 %)
Sexual exploitation	58 (49 %)	1 (4 %)	57 (63 %)
Unknown	32 (27 %)	18 (64 %)	14 (15 %)
Disorder			
Psychotic disorders	20 (17 %)	11 (39 %)	9 (10 %)
Other/psychological distress	99 (83 %)	17 (61 %)	82 (90 %)
Age median (range)	22 (8–49)	18 (8–44)	24 (10–49)
8–11	6 (5 %)	1 (4 %)	5 (5 %)
12–15	11 (9 %)	4 (14 %)	7 (7 %)
16–17	19 (16 %)	7 (25 %)	12 (13 %)
18–25	36 (30 %)	6 (21 %)	30 (33 %)
26–35	34 (29 %)	6 (21 %)	28 (31 %)
36+	13 (11 %)	4 (14 %)	9 (10 %)
Violence pre-trafficking			
Yes	57 (48 %)	11 (39 %)	46 (51 %)
No	62 (52 %)	17 (61 %)	45 (49 %)
Violence during trafficking			
Yes	69 (58 %)	8 (29 %)	61 (67 %)
No	50 (42 %)	20 (71 %)	30 (33 %)

Use of psychiatric services by the cohort and the cost of these services are presented in Table 2. Patient records documented 5,171 contacts with SLaM services during the study period, and the vast majority of these (92 %) were with outpatient SLaM services: 87 % (*n* = 103) of trafficked patients had at least one contact with an outpatient SLaM service, approximately a third had one or more inpatient stay (32 %, *n* = 38) and almost half (46 %, *n* = 55) had one or more emergency department contacts. Mean cost per person using each service was highest for inpatient stays.

**Table 2:** Total contacts and total costs by service type

	Inpatient	A&E	Outpatient
Total number of contacts	132	289	4750
Total number (%) of patients using the service	38 (32 %)	55 (46 %)	103 (87 %)
Mean cost (range) per person using the service (£)^a^	70,172 (445 to 617,658)	911 (162 to 7,472)	5,158 (59 to 44,775)

^a^For patients with at least one contact

**Fig. 1:**
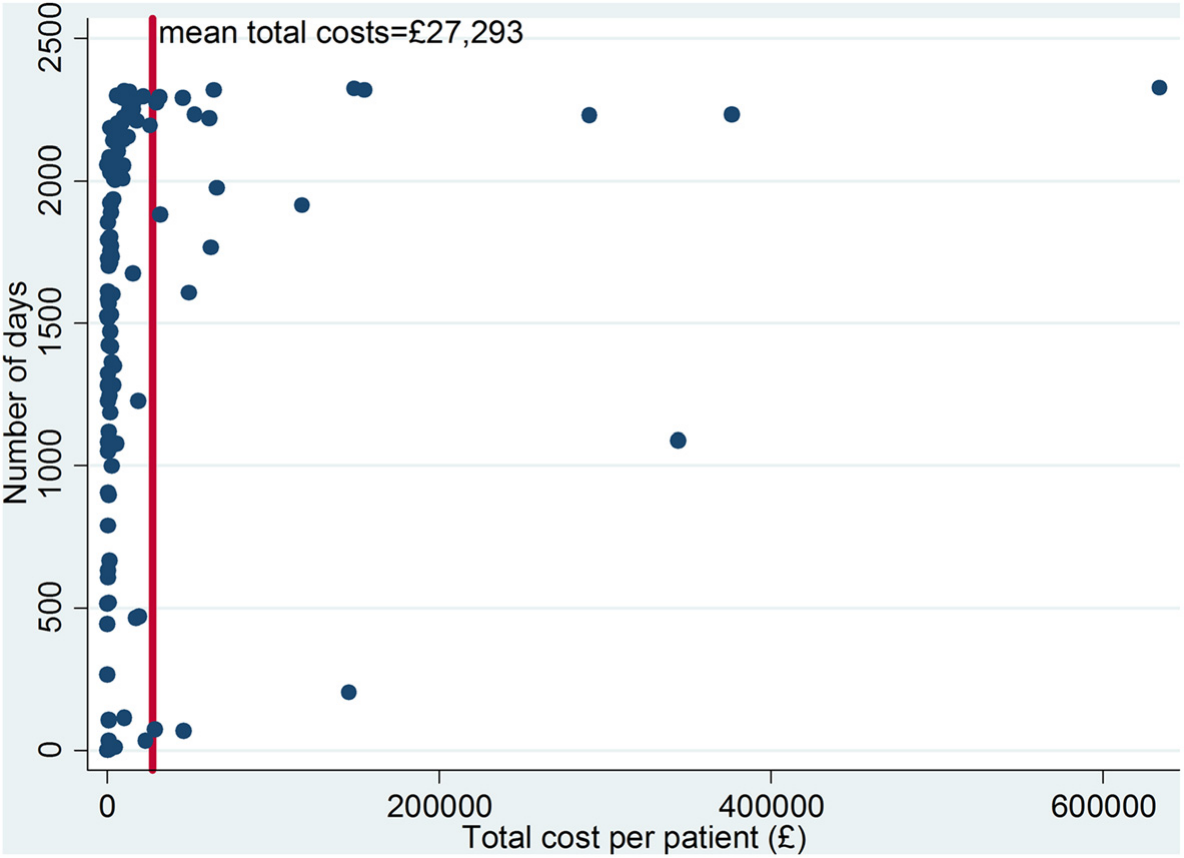
Scatter plot of total cost per patient by number of days patients were in contact with SLaM services

Total costs over the period for which trafficked patients were in contact with SLaM services are presented in Table 3. The mean duration of contact with services was approximately four years (1490 days, s.d. 757), with a mean total cost per patient of £27,293 (s.d. 80,985), or approximately £57 (s.d. 147) per patient per day. The distribution of the cost data in the cohort was positively skewed and thus the mean total costs are much higher than the median. This is illustrated in Fig. 1, which shows that only 20 patients had a total cost higher than the mean cost, with seven participants costing at least five times the mean cost.

**Table 3:** Total costs per patient

Variable	*N*	Mean (s.d.)^a^	Median	Min	Max
Number of days over which contacts took place	119	1490 (757)	1727	1	2325
Total cost per person (£)	119	27,293 (80,985)	3,366	59	633,970
Total cost per person per day (£)	119	57 (147)	3	0	725

^a^Please note that ${\displaystyle {\sum}_{i=0}^n\frac{\left(\frac{Total\kern0.5em  \cos ts}{number\kern0.5em  of\kern0.5em  days}\right)}{n}}\ne \frac{{\displaystyle {\sum}_{i=0}^n total\kern0.5em  cost/n}}{{\displaystyle {\sum}_{i=0}^n number\kern0.5em  of\kern0.5em  days/n}}$

Univariate associations between total mental health service costs per patient and key characteristics are shown in Table 4. Total mental health service costs per patient did not vary significantly according to type of exploitation, age at first contact with SLaM services or violence suffered during trafficking, and there was no difference comparing children (<18 years old) with adults (18 or over). Psychotic disorders were associated with higher total mental health service costs (*p* < 0.001) and documented history of pre-trafficking violence showed a weak association (*p* = 0.060). Costs were higher for men, who were more likely to have a diagnosis of psychotic disorder, than for women, although not significantly so (*p* = 0.091).

**Table 4:** Univariate associations with total cost per patient

Variable	*N*	Mean cost (£)	*P*-value
Type of exploitation			
Sexual	58	21,324	0.435
Other^a^	61	32,970	
Gender			
Male	28	49,899	0.091
Female	91	20,338	
Disorder			
Psychotic disorders^b^	20	98,452	0.000
Other/psychological distress^c^	99	12,918	
Age when admitted (split at median)			
≤ 22	60	32,058	0.612
> 22	59	22,447	
Violence pre-trafficking			
Yes	57	41,840	0.060
No	62	13,920	
Violence post-trafficking			
Yes	69	23,060	0.505
No	50	33,135	

^a^Financial, labour, domestic and unknown exploitation

^b^Bipolar and schizophrenia and other non-affective psychoses

^c^ Childhood emotional, depression, emotionally unstable PD, enduring personality change following catastrophic experience, mixed conduct disorder, OCD, PTSD, severe stress and adjustment, substance misuse, unspecified mental retardation, unspecified disorder of psychological development, psychological distress, and not assessed

Table 5 shows the results of the two multivariate models: model 1 containing only those variables found to be significantly associated with mental health service costs and model 2 containing all independent variables. Model selection did not alter the results; in both cases, psychotic disorders remained significant (*p* < 0.0001) and pre-trafficking violence became significant (*p* = 0.017 model 1 and 0.019 model 2). The results suggest that trafficked patients diagnosed with psychotic disorders cost approximately £33,000 more than trafficked patients with non-psychotic disorders or psychological distress and trafficked patients with a documented history of pre-trafficking violence cost approximately £90,000 more than trafficked patients who did not. Gender was not associated with total mental health service costs in the multivariate regression analysis and no other variables became significant. Results from bootstrap regression analyses and those based on generalised linear models were not substantially different from the OLS regression results reported in Table 5. Repeating our analyses using only the sample of adults aged 18 or over did not change these results and so are not presented here.

**Table 5:** Multivariate regression analysis for total cost per patient

Variable	Coefficient^a^ (95 % CI)	*p*-value
Model 1:		
Violence pre-trafficking (versus none)	32,635 (5,981 to 59,289)	0.017
Psychotic disorder (versus no other)	88,633 (53,024 to 124,242)	<0.0001
Adjusted *R* ^2^ = 0.198		
Model 2:		
Violence pre-trafficking (versus none)	32,643 (5,446 to 59,840)	0.019
Psychotic disorder (versus other)	93,939 (54,479 to 133,399)	<0.0001
Sexual exploitation (versus other)	17,171 (−17,728 to 52,071)	0.332
Gender female (versus male)	−14,105 (−51,701 to 23,490)	0.459
Age ≤22 (versus age >22)	18,437 (−10,176 to 47,051)	0.204
Violence post-trafficking (versus none)	5,954 (−24,869 to 36,778)	0.703
Adjusted *R* ^2^ = 0.218		

^a^The coefficient denotes the change in cost per unit increase in the variable measure

## Discussion

### Key findings

The study provides, for the first time, estimates of the use of secondary mental health services by trafficked people in England and the cost of secondary mental health care provision for this population. The mean duration of survivors’ contact with SLaM mental health services was four years, and the mean cost of care was £27,293 (s.d. 80,985). This figure, however, disguises substantial variation.

Two factors were identified as significant predictors of mental health service cost: diagnosis of psychotic disorder and documented history of pre-trafficking violence. Psychotic disorders were diagnosed in just under a fifth of the sample, and have been previously shown to be associated with more expensive mental health treatment [19]. Other disorders, including the more commonly diagnosed post-traumatic stress and depressive disorders were associated with significantly lower costs to services. Previous research has demonstrated an association between experiences of pre-trafficking violence and mental health problems among non-clinical samples of trafficked women recruited from post-trafficking support services [5, 15]. This is consistent with survey research suggesting that cumulative physical and sexual abuse is associated with a higher risk of mental disorder [20]. This study goes further by suggesting that among trafficking survivors with diagnosed mental disorders, those with experiences of pre-trafficking violence are likely to require more intensive mental health support. Neither gender nor type of exploitation was found to be associated with cost of mental health service provision.

Our finding that the majority of the sample were female and were trafficked for sexual exploitation is consistent with the national profile of identified cases of human trafficking during the period 2009–2012 (national statistics are not available for the period 2007–2008) [21]. In 2009, the UK introduced an identification and referral procedure to assess whether people were victims of human trafficking and therefore eligible for assistance. Between January 2009 and July 2012 there were 2,737 referrals: 70 % (*n* = 1918) of referrals were for females and 42 % (*n* = 1149) related to cases of sexual exploitation [21].

Due to resource limitations, we were not able to assess whether mental health service costs for trafficked people differ from those of non-trafficked patients. However, our previous finding that trafficked patients have a longer duration of inpatient admission and are more likely to be compulsorily admitted than matched non-trafficked patients suggests that costs may be higher for this patient group [6]. The mean duration of trafficked patients’ contact with secondary mental health services far exceeds the standard duration of support both in the UK and elsewhere [22, 23], and suggests there is a subgroup of trafficked people for whom long-term mental health, social, and welfare support will be vital. Yet, evidence on interventions to support the psychological recovery of trafficked people is lacking [24].

### Strengths and limitations

Psychiatric case registers which include complete electronic health records have exciting potential for estimating service use and associated costs for patients that are usually difficult to recruit into clinical studies. This study used an innovative data resource that allowed the searching and retrieval of anonymised full patient records for over 200,000 cases recorded on the SLaM Patient Journey System, a system in which data on gender, age, diagnosis, and mental health service use are routinely recorded. However, other key characteristics of interest for this study – including patients’ experiences of trafficking and experiences of violence – were not recorded in a standardised way and so could not be included or cannot be assumed to be entirely accurate [25].

All returned records were reviewed against the UN definition of human trafficking and against the study protocol, with an independent review of the first ten returned records and a random sample of a further 10 % of records by a second researcher. However, it is possible that patients inaccurately referred to as having experienced human trafficking by their care professionals may have been misclassified by the research team. A much larger number of trafficked patients are likely to not have been included in the sample because the professionals involved in their care were unaware that they have experienced trafficking or had not documented their concerns appropriately. In addition, pre-trafficking violence and violence during trafficking may not have been reported by all patients who disclosed they had been victims of trafficking, although the prevalence of pre-trafficking violence documented in the medical records of this sample is consistent with previous survey research with trafficked people [5, 15, 16], giving us some confidence in the rates recorded.

Cost estimates are limited to use of secondary mental health services, and do not include the use of other health services, including primary care, or the services provided by other sectors, such as Local Authority social services. Therefore, the cost results presented should be seen as a minimum for this population.

To our knowledge, there are no data on the number or characteristics of trafficked people in contact with mental health services elsewhere in England, and the generalizability of the findings beyond the study setting is unclear, including findings relating to characteristics predictive of higher cost. Further research in other settings is required.

## Conclusions

Mental health services are providing care for survivors of human trafficking, many of whom will require long-term mental health, social, and welfare support. Trafficked patients’ use of mental health services – and the cost of providing care– is highly variable, but patients with psychotic disorders and with experiences of pre-trafficking violence are likely to require more intensive support. Evidence is needed on the effectiveness of interventions to promote the recovery of survivors of trafficking.

## Abbreviations

CRIS, Case Register Interactive Search; ICD-10, International Classification of Diseases-10; NHS, National Health Service; PTSD, post-traumatic stress disorder; SD, standard deviation; SLaM, South London and Maudsley NHS Foundation Trust; UK, United Kingdom; UN, United Nations
